# The Impact of OnabotulinumtoxinA on Oral Pain Medication Prescription Fills and Low-Value Care in Patients with Cervical Dystonia in the United States: A Retrospective Claims Analysis

**DOI:** 10.3390/toxins18060269

**Published:** 2026-06-17

**Authors:** Christopher Rhyne, Annaliza Dominguez, Ning Cheng, Shivaji Manthena, Krutika Parikh, Bahman Jabbari

**Affiliations:** 1 Norton Neuroscience Institute, Louisville, KY 40202, USA; 2 AbbVie Inc., North Chicago, IL 60064, USA; 3 Department of Neurology, Yale School of Medicine, New Haven, CT 06510, USA

**Keywords:** cervical dystonia, pain, onabotulinumtoxinA, opioid, skeletal muscle relaxant, benzodiazepine

## Abstract

(1) Background: While evidence supporting the use of onabotulinumtoxinA in patients with cervical dystonia (CD) is well-established, more evidence is needed to understand if onabotulinumtoxinA treatment can reduce reliance on addictive medication. (2) Methods: This retrospective study used claims data to evaluate the impact of onabotulinumtoxinA treatment on opioid, benzodiazepine (BZD), and skeletal muscle relaxant (SMR) prescription fills among patients diagnosed with CD with prior use of these medications. Among the 564 eligible patients, three non-mutually exclusive cohorts were identified based on medication use data: opioid (*n* = 306), BZD (*n* = 271), and SMR (*n* = 371). The follow-up period continued for 12 months after onabotulinumtoxinA treatment initiation. (3) Results: Among patients with >1 opioid fill at baseline, 30.4% had no opioid fills in the 12-month period following onabotulinumtoxinA initiation. The opioid cohort had significantly reduced mean opioid prescription fills per patient (17.0%; *p* < 0.0001) in the follow-up period compared with baseline. A 30.0% decrease was observed in the mean morphine milligram equivalent (MME)/day after onabotulinumtoxinA initiation (*p* < 0.0001). Similar trends of decreased BZD and SMR prescription fills were observed. (4) Conclusions: Following initiation of onabotulinumtoxinA treatment, patients living with CD who had prior opioid, BZD, or SMR use had significant reductions in respective prescription fills for opioids, BZDs, and SMRs.

## 1. Introduction

Cervical dystonia (CD), a neurological condition characterized by involuntary contraction of neck and/or shoulder muscles, can cause abnormal posture as well as twisting and repetitive movements frequently accompanied by pain [1]. The heterogeneous clinical presentation of CD and the lack of validated clinical diagnostic guidelines contribute to underdiagnosis or misdiagnosis [2,3]. Prevalence varies widely, with reported rates up to 2800 cases per million in the United States (US) and higher incidence rates among women between 40 and 60 years of age [2,3,4]. Most patients seek treatment for CD due to chronic pain severely burdening their quality of life, including widespread economic impacts with reduced productivity reported in approximately 60% of patients and loss of employment reported in an estimated 18.9–38.5% of patients [5,6,7].

General practitioners may focus on pain management for patients rather than treating the underlying causes [8], especially as the brain’s natural pain-control mechanisms may not function properly in this population [9,10]. Opioids are more likely to be prescribed for patients with a greater severity of CD symptoms. However, these medications have an elevated potential for addiction, with a multisite study concluding that 11% of patients with CD met the diagnostic criteria for substance abuse and that 26% of these patients were prescribed opioids [11]. Benzodiazepines (BZDs) may be prescribed to help reduce neural overactivity; however, somnolence, dizziness, and behavioral abnormalities may deter patients from long-term use [12]. Skeletal muscle relaxants (SMRs) may be prescribed to provide modest alleviation of pain associated with muscle spasms [13]. Payers may designate these medications as low-value care, which includes services with minimal clinical benefit or those that have the potential to cause harm when used as part of long-term care for managing chronic symptoms of CD [14].

In patients with CD, particularly older adults with polypharmacy, prolonged use of opioids, BZDs, and SMRs significantly increases the risk of falls, cognitive impairment, and dependency that may outweigh symptomatic benefits [14]. The US Centers for Medicare and Medicaid Services (CMS) developed standards to promote high-quality outcomes and safe care practices for patients. These CMS national standards include quality measures and Part D policies intended to explicitly target high-risk prescribing patterns, including overutilization of opioids and central nervous system depressant medications (e.g., BZDs), specifically emphasizing the dangers of overdose with concomitant use [15].

Low-value care can be reduced through evidence-based standard of care interventions, such as botulinum toxins, which offer both superior clinical outcomes and improved safety profiles by targeting the specific muscles affected rather than through systemic effects [16,17,18,19]. Treatments with longer-lasting therapeutic effects are critical in helping reduce the substantial healthcare cost burden of CD; notably, a recent study estimated that nearly all (96.1%) patients with CD had at least one prescription fill (mean 18.4 fills) preceding initiation of treatment with botulinum toxins type A [20]. The coverage of botulinum toxin type A is enabled under CMS local coverage determination (LCD) policies and may reduce downstream reliance on opioids in patients living with CD [15]. Currently, five botulinum toxin treatments are approved by the US Food and Drug Administration (FDA) for treating CD, including onabotulinumtoxinA, incobotulinumtoxinA, abobotulinumtoxinA, daxibotulinumtoxinA, and rimabotulinumtoxinB [21,22,23]. As the first botulinum toxin approved by the FDA in 2000 for treatment of CD [24,25], onabotulinumtoxinA (AbbVie Inc., North Chicago, IL, USA) has a particularly strong and long-standing body of evidence demonstrating sustained efficacy, safety, and tolerability [22] across multiple subtypes of CD [26], and thus is the primary focus of this study.

Evidence supporting the use of onabotulinumtoxinA in patients with CD is well established for the reduction of pain and other symptoms. However, the real-world utilization patterns and downstream medication burden at the population level have not been characterized using large-scale claims data to determine whether onabotulinumtoxinA treatment can reduce reliance on addictive medications like opioids, alongside other low-value care therapies like BZDs and SMRs. To our knowledge, this is the first retrospective cohort study using real-world claims data to investigate the impact of onabotulinumtoxinA treatment on oral opioid, BZD, and SMR prescription fills among patients diagnosed with CD and with prior use of these medications.

## 2. Results

### 2.1. Patient Demographics and Clinical Characteristics

The mean age across all patients (*N* = 564) was 58.1 ± 14.3 years, with a majority of patients being female (71.6%) and white (64.9%). Patient demographics and baseline characteristics were similar across the non-mutually exclusive opioid (*n* = 306), BZD (*n* = 271), and SMR (*n* = 371) cohorts (Table 1). Most patients across all cohorts had baseline pain, including pain located in the back, neck, or related to fibromyalgia (Table 1). Anxiety, the second-most common comorbidity across all cohorts, was present in the majority of patients in the BZD cohort (Table 1). The mean time from CD diagnosis to onabotulinumtoxinA initiation ranged from 6 to 8 months; 7.8 ± 6.0 months for the opioid cohort; 5.9 ± 6.7 months for the BZD cohort; and 6.0 ± 8.0 months for the SMR cohort.

### 2.2. Pain Medication Prescription Fills Following OnabotulinumtoxinA Initiation

#### 2.2.1. Decrease in Opioid Fills Following OnabotulinumtoxinA Initiation

**Figure 1:**
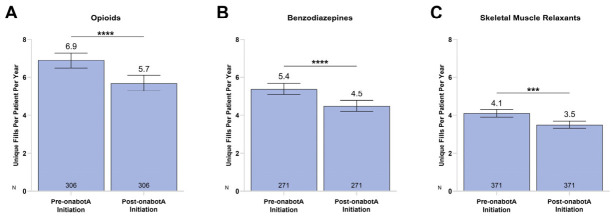
Pre- and post-initiation prescription fills with onabotulinumtoxinA: (**A**) Opioid, (**B**) benzodiazepine, and (**C**) skeletal muscle relaxant mean prescription fills. Abbreviations: onabotA, onabotulinumtoxinA. Error bars represent standard errors of the mean. *** *p* < 0.001, **** *p* < 0.0001.

**Figure 2:**
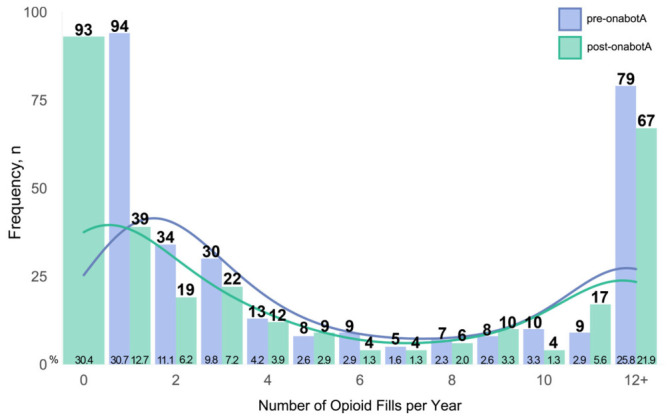
Histogram of mean annual opioid fills with overlaid density curves, stratified by pre- and post-onabotA periods. Abbreviations: onabotA, onabotulinumtoxinA.

Within the opioid cohort, mean opioid prescription fills per patient significantly decreased by 17.0% in the 12 months following onabotulinumtoxinA initiation (5.7 ± 7.0 mean post-initiation fills vs. 6.9 ± 7.2 mean pre-initiation fills; *p* < 0.0001; Figure 1A). There was a general downward shift in the number of annual opioid prescription fills following onabotulinumtoxinA initiation (Figure 2). Approximately one-third (30.4%) of patients with opioid use prior to initiation of onabotulinumtoxinA treatment had 0 opioid fills in the 12 months following initiation. Additionally, the proportion of patients with ≥12 opioid fills per year significantly decreased by 5.5% (*p* = 0.002).

**Figure 3:**
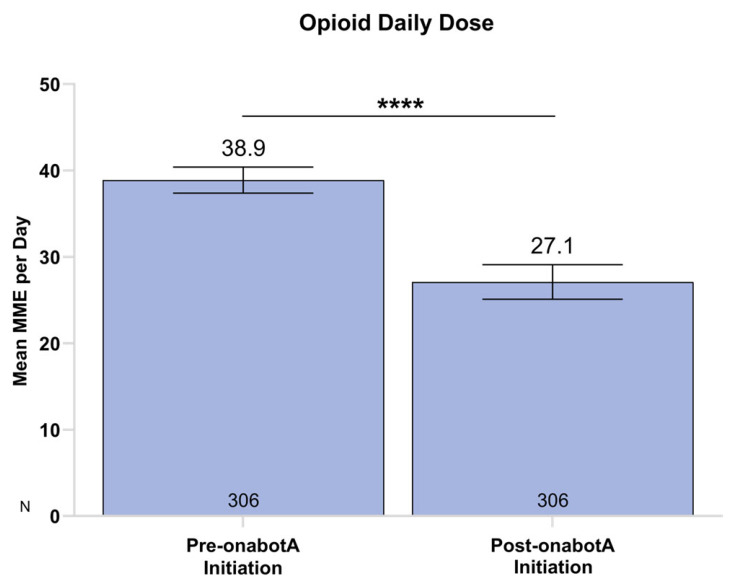
Change in mean MME/day per patient. MME/day (morphine milligram equivalents) refers to the total daily dosage of opioid medications a person takes, expressed in terms of the equivalent amount of morphine for standardization. Abbreviations: onabotA, onabotulinumtoxinA. Error bars represent standard errors of the mean. **** *p* < 0.0001.

The calculated mean daily opioid dose per patient in the opioid cohort significantly decreased by 30.3% in the 12 months following onabotulinumtoxinA initiation (38.9 vs. 27.1 mean morphine milligram equivalent [MME]/day, *p* < 0.0001; Figure 3).

#### 2.2.2. Decrease in BZD Fills Following OnabotulinumtoxinA Initiation

**Figure 4:**
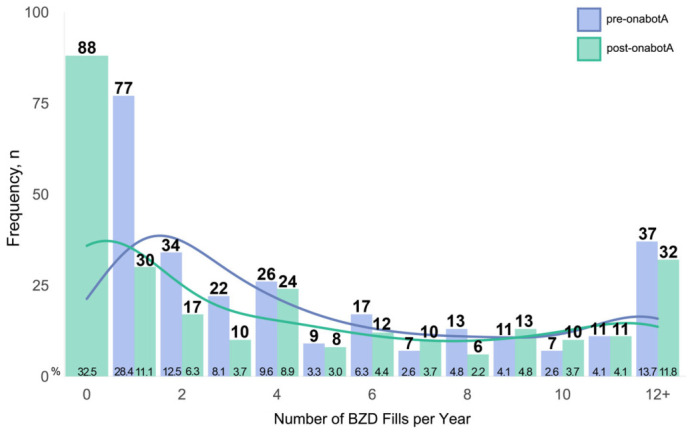
Histogram of mean annual BZD fills with overlaid density curves, stratified by pre- and post-onabotA periods. Abbreviations: BZD, benzodiazepine; onabotA, onabotulinumtoxinA.

Within the BZD cohort, mean BZD prescription fills per patient significantly decreased by 17.0% in the 12 months following onabotulinumtoxinA initiation (4.5 ± 5.4 post-initiation fills vs. 5.4 ± 5.2 pre-initiation fills, *p* < 0.0001; Figure 1B). In the 12 months following onabotulinumtoxinA initiation, 32.5% of patients with prior BZD use had 0 BZD prescription fills (Figure 4). Additionally, the proportion of patients with ≤6 BZD fills significantly decreased following initiation of onabotulinumtoxinA treatment (*p* ≤ 0.01).

#### 2.2.3. Decrease in SMR Fills Following OnabotulinumtoxinA Initiation

**Figure 5:**
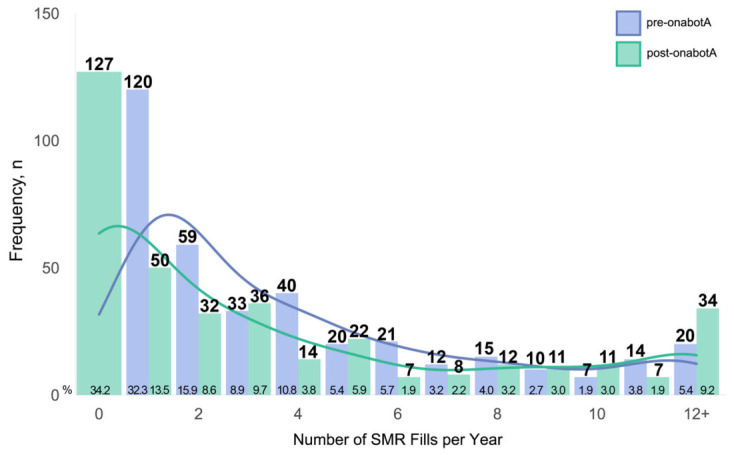
Histogram of mean annual SMR fills with overlaid density curves, stratified by pre- and post-onabotA periods. Abbreviations: onabotA, onabotulinumtoxinA; SMR, skeletal muscle relaxant.

Within the SMR cohort, mean SMR prescription fills per patient significantly decreased by 14.0% in the 12 months following onabotulinumtoxinA initiation (3.5 ± 4.3 post-initiation fills vs. 4.1 ± 3.7 pre-initiation fills; *p* < 0.001; Figure 1C). In the 12 months following the initiation of onabotulinumtoxinA treatment, 34.2% of patients with prior SMR use had 0 SMR prescription fills (Figure 5). Additionally, the proportion of patients with ≤6 SMR fills significantly decreased (*p* < 0.001).

## 3. Discussion

Patients with CD and prior opioid use had significant decreases in mean opioid prescription fills as well as opioid daily doses during the 12-month period following initiation of onabotulinumtoxinA treatment. Notably, nearly one-third of patients had no opioid fills after receiving onabotulinumtoxinA, and the proportion of chronic opioid users with 12 or more opioid fills was significantly reduced. Patients with CD and prior BZD or SMR use similarly had significant decreases in prescription fills of these medications during the 12-month period following initiation of onabotulinumtoxinA treatment. These findings suggest that onabotulinumtoxinA treatment may reduce reliance on these low-value oral prescriptions in patients with CD.

As these cohorts were non-mutually exclusive, patients with CD who decreased their fills of oral opioids may have switched to oral SMRs or other less-addictive medications, although this study design did not assess switching between medications. SMR usage across the general population has increased, with a nationwide survey reporting that physician prescriptions of SMRs nearly doubled from 2005 to 2016 [27]. The use of long-term SMRs may be beneficial for managing muscle spasms, cramps, and neck pain [28], which commonly present in patients with CD. Clinicians may be willing to switch patients to less-addictive medications using planned tapering and a shared decision-making model [29].

The reduced fills of prescription analgesics in patients with CD suggest that treatment with onabotulinumtoxinA may have reduced these patients’ pain. Real-world data from a multisite study indicated that patients with CD reported significant improvement across all participant-assessed Cervical Dystonia Impact Profile (CDIP)-58 subscales, as well as significant reduction in pain as assessed using the Pain Numeric Rating Scale [7]. With an estimated 55–90% of patients with CD affected by pain [10,30,31,32], the reduction in prescription analgesic fills supports important efforts related to deprescribing and reducing the risks associated with adverse effects and polypharmacy.

The significant reduction in opioid fills, including the large percentage of patients who stopped filling opioid prescriptions, suggests that onabotulinumtoxinA treatment may help reduce low-value care for patients with CD. By reducing high-risk prescribing and mitigating harms related to opioids, treatment of CD using onabotulinumtoxinA aligns with the efforts made by CMS to improve the safety of pain management. Rather than relying on systemic analgesics, targeting underlying pathophysiology may enhance patient safety while also improving their quality of life. Prior research has shown that patients with moderate-to-severe CD have significantly increased absenteeism and presenteeism due to their pain, and treatment of these patients with onabotulinumtoxinA may help improve CD-related impacts on employment [32]. Addressing the cost and travel barriers that discourage access to botulinum neurotoxin treatments may improve patient adherence [33]. To this end, evolving CMS guidance reduces barriers to treatment access to improve outcomes and safety for patients with CD.

Several limitations should be considered when interpreting these study findings. The reasons for opioid, BZD, or SMR fills were not captured within the claims and therefore cannot be attributed to the management of CD symptoms. In addition, neither pain status nor the severity of pain was captured within the claims database. Due to the observational nature of the study design, causality cannot be established between onabotulinumtoxinA usage and the reduction in pain medication fills. Although prescription fills served as a proxy for medication exposure, this study was not designed to confirm actual consumption nor to capture medication switches over the observed treatment course. Additionally, patients who were uninsured, covered under Medicare fee-for-service, covered by Medicaid, or covered commercially through a payer that does not provide claims data to Optum^®^ Clinformatics^®^ Data Mart Database (CDM) were not captured within this dataset. Therefore, these findings may not be generalizable to all CD patients within the US due to differences in care access, prescribing patterns, and demographic characteristics across coverage types. Due to the descriptive nature of the analyses performed, the potential for residual confounding remains across factors such as age, biological sex, comorbidity burden, and baseline medication use. These findings represent associations which may be influenced by coding limitations and unmeasured clinical variables inherent to administrative claims data.

The design of this study did not track switches from one medication to another over the observed treatment course; however, future studies may investigate whether patients were switched from opioid medications to less-addictive alternatives. While this analysis was conducted within the scope of available claims data, future work may expand upon these pilot findings to evaluate the impact of CD subtype or severity, onabotulinumtoxinA dosing, the use of guidance imaging (e.g., ultrasound or electromyography) during administration, or whether patients also underwent physiotherapy during the study period; all of which may influence pain-related outcomes [34,35,36,37]. Additionally, assessment of clinical parameters using validated pain scales and patient-reported outcome measures related to quality of life would be valuable to address gaps in the literature [21,37]. A comparison with other botulinum toxins would also help to elucidate whether similar prescription fill patterns are observed across botulinum toxin treatments. Future research may also examine whether CD treatment using onabotulinumtoxinA disrupts opioid polypharmacy inertia, a phenomenon wherein patients prescribed opioids are more likely to receive additional unique opioid prescriptions over time. Further research may also evaluate the impact of time to onabotulinumtoxinA initiation in reducing total opioid use and opioid regimen complexity, which may suggest a circuit-breaking role of onabotulinumtoxinA in the feedforward cycle of opioid accumulation for patients with CD.

## 4. Conclusions

Following initiation of onabotulinumtoxinA treatment, patients living with CD who had prior opioid, BZD, or SMR use had significant reductions in respective prescription fills for opioids, BZDs, and SMRs. Nearly one-third of patients with CD and prior opioid use had no opioid prescription fills for 12 months after receiving onabotulinumtoxinA.

## 5. Materials and Methods

### 5.1. Study Design

This retrospective cohort study compared oral opioid, oral BZD, and oral SMR use among eligible patients diagnosed with CD—defined by International Classification of Diseases, 10th Revision, Clinical Modification (ICD-10-CM) diagnosis code (G24.3)—in the US using claims data during the 12 months prior to onabotulinumtoxinA treatment initiation (i.e., pre-index; index date, date of the first medical claim of onabotulinumtoxinA administration) (Figure S1, Supplementary Materials). The follow-up period continued for 12 months after the index date. Changes in opioid, BZD, and SMR utilization were captured via pre- vs. post-index differences in mean prescription fills per patient and mean opioid daily dose per patient (MME/day) [38] assessed in respective cohorts using data from the Optum^®^ CDM from 1 January 2017 to 31 December 2023.

To be eligible for inclusion in this study, patients had to have a diagnosis of CD; ≥1 pharmacy claim for an opioid, BZD, or SMR; and ≥1 medical claim for onabotulinumtoxinA administration. All opioid, BZD, and SMR prescriptions analyzed were oral medications with the exception of fentanyl (opioid category), which may also be administered via nasal route or patch. Detailed inclusion and exclusion criteria are presented in Figure S2 and Table S1 (Supplementary Materials). The primary outcomes compared the mean change in opioid fills (Table S2, Supplementary Materials), the number of opioid fills per patient, and the change in opioid daily dose (MME/day) per patient between the 12-month baseline and the 12 month follow-up period for the opioid cohort. The secondary outcomes for the BZD and SMR cohorts followed similar comparisons assessing the mean change in and the number of respective BZD and SMR fills (Table S2, Supplementary Materials).

### 5.2. Data Source

This study used claims data from Optum^®^ CDM (https://business.optum.com/en/data-analytics/life-sciences/real-world-data/claims-data.html [accessed 1 September 2025]), which is a large administrative health claims database for members of large commercial and Medicare Advantage health plans, covering approximately 15–19 million annual lives of United Health Group members in the US. The CDM database uses medical and pharmacy claims to derive patient-level enrollment information, health care costs, and resource utilization information. CDM administrative claims submitted for payment by providers and pharmacies are verified, adjudicated, and de-identified prior to inclusion. Data are de-identified and compliant with the Health Insurance Portability and Accountability Act (HIPAA).

### 5.3. Statistical Analysis

Descriptive statistics were used to summarize patient characteristics and outcomes. Mean and standard deviation (SD; indicated by ±) were used for continuous variables. Counts and percentages were used for categorical variables. Histograms and related frequency distribution curves were plotted to show the changes in number of annual mean drug prescription fills for opioids, BZDs, and SMRs.

To compare opioid, BZD, and SMR prescription fills pre- and post- index onabotulinumtoxinA initiation, different statistical methods were used according to variable distributions. Paired *t*-tests were used for continuous variables that were normally distributed and Wilcoxon signed-rank tests were used for variables that were not normally distributed. Pre- and post-differences in categorical measures were evaluated via McNemar’s tests. No imputations for missing data were performed.

## Figures and Tables

**Table 1:** Patient demographics and baseline clinical characteristics.

Characteristic	Opioid Cohort (*n* = 306)	SMR Cohort (*n* = 371)	BZD Cohort (*n* = 271)
Age (years), mean (SD)	59.3 (13.4)	56.2 (14.2)	58.4 (13.8)
Sex, female, *n* (%)	215 (70.3)	271 (73.0)	205 (75.6)
Race/Ethnicity, *n* (%)			
White	219 (71.6)	239 (64.4)	180 (66.4)
Black	13 (4.2)	15 (4.0)	7 (2.6)
Asian or Pacific Islander	3 (1.0)	6 (1.6)	5 (1.8)
Hispanic or Latino	0 (0.0)	0 (0.0)	0 (0.0)
Other or Unknown	71 (23.2)	111 (29.9)	79 (29.2)
Region, *n* (%)			
South	118 (38.6)	131 (35.3)	94 (34.7)
West	95 (31.0)	98 (26.4)	80 (29.5)
Midwest	64 (20.9)	90 (24.3)	63 (23.2)
Northeast	29 (9.5)	52 (14.0)	34 (12.5)
Payer, *n* (%)			
Medicare	162 (52.9)	157 (42.3)	123 (45.4)
Commercial	144 (47.1)	214 (57.7)	147 (54.2)
Unknown	0 (0.0)	0 (0.0)	1 (0.4)
Charlson-Quan Comorbidity Index, mean (SD)	0.8 (1.3)	0.6 (1.2)	0.6 (1.1)
Other comorbidities, *n* (%)			
Pain ^a^	250 (81.7)	298 (80.3)	199 (73.4)
Anxiety	107 (35.0)	130 (35.0)	142 (52.4)
Depression	105 (34.3)	118 (31.8)	98 (36.2)
Sleep disorders	84 (27.5)	82 (22.1)	77 (28.4)
Headache	28 (9.2)	30 (8.1)	25 (9.2)
Mood disorders	8 (2.6)	8 (2.2)	7 (2.6)
Epilepsy and recurrent seizures	4 (1.3)	8 (2.2)	5 (1.8)
Suicidal ideation	2 (0.7)	3 (0.8)	2 (0.7)
Time from CD diagnosis to onabotulinumtoxinA start (months), mean (SD)	7.8 (6)	6.0 (8)	5.9 (6.7)
Baseline CD treatments other than opioids, BZDs, or SMRs, *n* (%)			
Total	157 (51.3)	167 (45.0)	109 (40.2)
NSAIDs	149 (48.7)	160 (43.1)	100 (36.9)
Anticholinergics ^b^	10 (3.3)	11 (3.0)	12 (4.4)
Deep brain stimulation	1 (0.3)	0 (0.0)	1 (0.4)
Intrathecal baclofen	0 (0.0)	0 (0.0)	0 (0.0)

^a^ Includes back pain, neck pain, fibromyalgia. ^b^ Trihexyphenidyl/Other. Abbreviations: BZD, benzodiazepine; CD, cervical dystonia; NSAIDs, nonsteroidal anti-inflammatory drugs; SD, standard deviation; SMR, skeletal muscle relaxant.

## Data Availability

The original contributions presented in this study are included in the article/Supplementary Material. Further inquiries can be directed to the corresponding author.

## Supplementary Materials

The following supporting information can be downloaded at: https://www.mdpi.com/article/10.3390/toxins18060269/s1, Figure S1: Study schema; Figure S2: Study attrition funnel; Table S1: Study eligibility criteria; Table S2: Included oral opioids, oral BZDs, and oral SMRs.
