# Supplementary material for: The Impact of OnabotulinumtoxinA on Oral Pain Medication Prescription Fills and Low-Value Care in Patients with Cervical Dystonia in the United States: A Retrospective Claims Analysis
Source: Toxins (Basel). 2026 Jun 17;18(6):269. doi: 10.3390/toxins18060269 (PMC13307583; doi:10.3390/toxins18060269)
Supplement: Supplementary file 1 [file toxins-18-00269-s001.zip › toxins-4202524-supplementary.pdf]

Supplementary Materials: The Impact of OnabotulinumtoxinA on Oral Pain Medication Prescription Fills and Low-Value Care in Patients with Cervical Dystonia in the United States: A Retrospective Claims Analysis

Christopher Rhyne, Annaliza Dominguez, Ning Cheng, Shivaji Manthena, Krutika Parikh and Bahman Jabbari

Table S1. Study eligibility criteria.

Eligibility type	Criteria
**Inclusion**	• ≥1 inpatient or ≥2 outpatient medical claims with ICD-10-CM diagnosis of CD during the study period from January 1, 2017 to December 31, 2022[a]<br>• ≥1 pharmacy claims for an oral opioid, BZD, SMR during the identification period<br>• ≥1 medical claims for a onabotulinumtoxinA administration identified by HCPCS codes on or after CD diagnosis date[b]<br>• Continuous medical and pharmacy insurance coverage between the CD diagnosis date and index date<br>• ≥12 months of continuous medical and pharmacy insurance coverage before (exclusive) the index date (baseline period); a gap of no greater than 30 days is allowed<br>• ≥12 months of continuous medical and pharmacy insurance coverage after (exclusive) the index date (follow-up period); a gap of no greater than 30 days is allowed<br>• ≥18 years of age at the index date<br>• Inclusion in the opioid, SMR, and BZD cohorts requires ≥1 pharmacy claim for an oral opioid, oral SMR, or BZD, respectively, during the pre-index period; cohorts may not be mutually exclusive
**Exclusion**	• Diagnosis of cancer or malignancy during the study period<br>• ≥1 medical (by HCPCS) or pharmacy (by NDC) claims for onabotulinumtoxinA administration prior to the CD diagnosis date<br>• ≥1 (by HCPCS) claims for onabotulinumtoxinA with a diagnosis claim for a onabotulinumtoxinA indication (chronic migraine, spasticity, overactive bladder, neurogenic detrusor overactivity, severe axillary hyperhidrosis, blepharospasm, or strabismus) other than CD at any time during the study period<br>• ≥1 medical (by HCPCS) or pharmacy (by NDC) claims for toxins other than onabotulinumtoxinA at any time during the study period

[a] The first observed CD diagnosis defines the CD diagnosis date.

[b] The date of the first medical claim of onabotulinumtoxinA administration defines the index date.

Abbreviations: BZD, benzodiazepine; CD, cervical dystonia; HCPCS, Healthcare Common Procedure Coding System; ICD-10-CM, International Classification of Diseases, Tenth Revision, Clinical Modification; NDC, National Drug Code; SMR, skeletal muscle relaxant.

Table S2. Included opioids, SMRs, and BZDs.

Opioid	SMR	BZD
• Codeine		• Alprazolam
• Dihydrocodeine		• Clonazepam
• Fentanyl*		• Chlordiazepoxide
• Hydrocodone	• Cyclobenzaprine	• Clorazepate
• Hydromorphone	• Tizanidine	• Diazepam
• Levorphanol	• Oral Baclofen	• Estazolam
• Methadone	• Dantrolene	• Flurazepam
• Meperidine	• Carisoprodol	• Lorazepam
• Morphine	• Chlorzoxazone	• Midazolam
• Oxycodone	• Metaxalone	• Oxazepam
• Oxymorphone	• Methocarbamol	• Quazepam
• Pentazocine	• Orphenadrine	• Temazepam
• Tapentadol		• Triazolam
• Tramadol		

*All prescriptions are administered orally, except for fentanyl (opioid), which is also available in patch and nasal forms, as part of the official MME table.

Abbreviations: BZD, benzodiazepine; SMR, skeletal muscle relaxant.

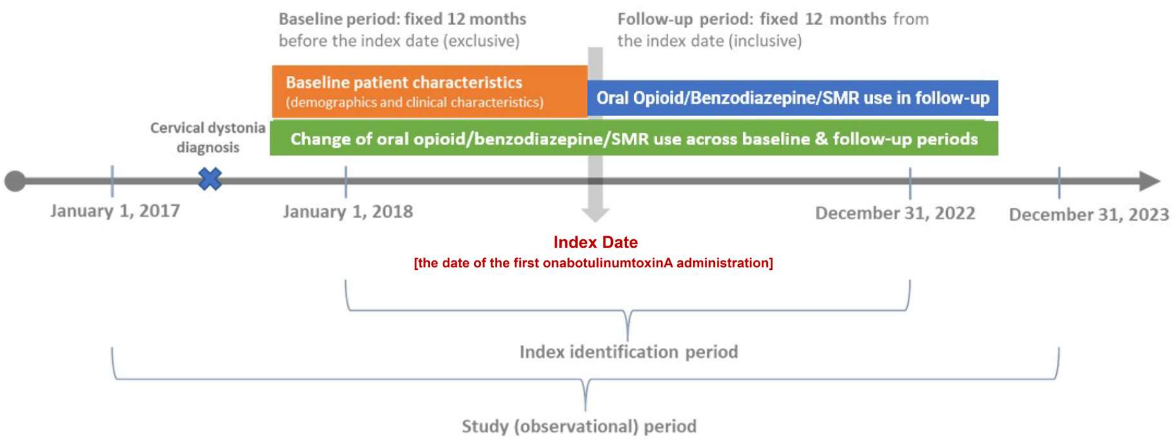

Figure S1. Study schema. Abbreviation: SMR, skeletal muscle relaxant.

**≥1 inpatient or ≥2 outpatient CD diagnoses between January 1, 2017 - December 31, 2023**
n=27,956
**≥1 medical claim for onabotA administration (index date) on or after CD diagnosis and ≥18 years old at index**
n=9,863
**≥12 months' continuous enrollment (pharmacy and medical) pre- and post-index**
n=5,061
**No claim for onabotA prior to CD diagnosis**
n=3,433
**None of the following during the study period: diagnosis claim for an onabotA indication other than CD, claim for other botulinum toxins, or cancer diagnosis**
n=714
**≥1 prescription claim for opioid, BZD, or SMR during study period**
n=564

Figure S2. Study attrition funnel. Abbreviations: BZD, benzodiazepine; CD, cervical dystonia; SMR, skeletal muscle relaxant.
